# Experimental Study on Acoustic Emission Features and Energy Dissipation Properties during Rock Shear-Slip Process

**DOI:** 10.3390/ma17194684

**Published:** 2024-09-24

**Authors:** Zhengnan Zhang, Xiangxin Liu, Kui Zhao, Zhengzhao Liang, Bin Gong, Xun You

**Affiliations:** 1 School of Resource and Environmental Engineering, Jiangxi University of Science and Technology, Ganzhou 341000, China; 6120220370@mail.jxust.edu.cn (Z.Z.); kuizhao@jxust.edu.cn (K.Z.); 13635551658@163.com (X.Y.); 2 School of Civil and Surveying & Mapping Engineering, Jiangxi University of Science and Technology, Ganzhou 341000, China; 3 State Key Laboratory of Coastal & Offshore Engineering, Dalian University of Technology, Dalian 116024, China; liangzz@dlut.edu.cn; 4 Department of Civil and Environmental Engineering, Brunel University London, London UB8 3PH, UK; bin.gong@brunel.ac.uk

**Keywords:** rock mechanics, shear-slip, acoustic emission (AE), energy dissipation, fracture type

## Abstract

The features of rock shear-slip fracturing are closely related to the stability of rock mass engineering. Granite, white sandstone, red sandstone, and yellow sandstone specimens were selected in this study. The loading phase of “shear failure > slow slip > fast slip” was set up to explore the correlation between fracture type, acoustic emission (AE) features, and energy dissipation during the rock fracturing process. The results show that there is a strong correlation between fracture type, energy dissipation, and AE features. The energy dissipation ratio of tension-shear (T-S) composite, shear, and tensile types is 10:100:1. The fracture types in the shear failure phase are mainly tensile and TS composite types. The differential mechanism of energy dissipation of different rocks during the shear-slip process is revealed from the physical property perspectives of mineral composition, particle size, and diagenetic mode. These results provide a necessary research basis for energy dissipation research in rock failure and offer an important scientific foundation for analyzing the fracture propagation problem in the shear-slip process. They also provide a research basis for further understanding the acoustic emission characteristics and crack type evolution during rock shear and slip processes, which helps to better understand the shear failure mechanism of natural joints and provides a reference for the identification of precursors of shear disasters in geotechnical engineering.

## 1. Introduction

Shear or shear-slip types are common failure modes of rock mass engineering, such as shear-slip failure of rock slope, structural plane slip, shear-slip rock pillar failure, etc. These failure modes all exhibit typical gradual failure characteristics, corresponding to the gradual weakening of rock mechanical properties [1,2]. Non-uniformity and anisotropy are the internal causes of crack initiation, while external stress is the external cause of crack propagation [3,4].

To explore the law of rock shear fracture evolution, Liu et al. [5] conducted rock shear AE monitoring tests, and found that the fracture type and the fracture length distribution were determined by the normal stress level. Wang et al. [6] indicated that the shear stress, the slip rate, and the stress drop in shear-slip events were significantly higher than those in low-stress states and that the timing and type of main failure in the shearing process will change when normal stress increases. Guo et al. [7] used an improved smooth joint model combined with the contact discrimination method of particle groups on both sides of the structural plane to reveal three shear mechanisms: sliding, wearing, and shearing. Zhai et al. [8] analyzed the influence of shear rate on the fracturing process of layered samples with different orientations based on the direct shear performance. The shear rate was positively correlated with the proportion of high-energy AE events. In triaxial shear experiments, confining pressure has a significant impact on the stress threshold and failure characteristics of the shear slip process, and this impact increases with the transition of the shear slip stage [9]. Xin et al. [10] found that the conversion rules between the elastic strain energy and the dissipated strain energy before and after peak loading are different.

As a nondestructive testing technology, acoustic emission detection is an important technical means to study the crack propagation and failure modes of rocks under different loads [11]. Acoustic emission parameters not only reflect the mechanical properties of rock but also contain information about the evolution of internal damage in rock [12]. The crack propagation and penetration during the process of rock failure can lead to various characteristics in acoustic emission activity. Du et al. [13] used parameters such as peak AE frequency, AF/RA (RA, Rise time/Amplitude; AF, Average/Frequency), energy, and others to identify rock fracture modes and fracture types; AE characteristics were explored the crack propagation and crack mode. Wang et al. [14] found out that b-value from continuous decline to sudden decline is an effective law for identifying the precursors of rock instability.

To investigate the influence of different dividing lines of AF/RA on the differentiation of fracture modes, Wang et al. [15] utilized the Kneedle algorithm to establish the discriminant equation for distinguishing rock fracture modes. Zhan et al. [16] conducted uniaxial compression tests by marble, fine-grained granite, diorite, and coarse-grained granite specimens, and identified the optimal ratio of RA to AF in the analysis of AE parameters through the statistical analysis of main frequency features. Gong et al. [17] defined the stress corresponding to the rapid growth time point of the cumulative shear source as the initiation stress. Liu et al. [18] applied AF/RA parameters to the support vector machine algorithm to differentiate between tensile and shear fractures. The proportion changes of tensile microcracks and shear microcracks in different stages of the mechanical curve of rock salt under uniaxial compression can also be obtained through the AF-RA relationship [19].

Previous studies have shown that the AF/RA parameter is widely used to distinguish fracture types. However, it is necessary to further explore the relationship between the fracture type and energy dissipation from a quantitative perspective. In this paper, a shear-slip AE monitoring test is conducted using non-penetrating joint rock specimens, and the loading path of “shear failure > slow slip > fast slip” is set according to the failure process of “stable > shear failure > slip > instability” of an underground pillar rock mass. The quantitative relationship between fracture type and energy dissipation of different rocks is examined and the mechanism of energy dissipation in various rock failure processes is elucidated.

## 2. Rock Shear-Slip Test

### 2.1. Rock Specimens

**Figure 1:**
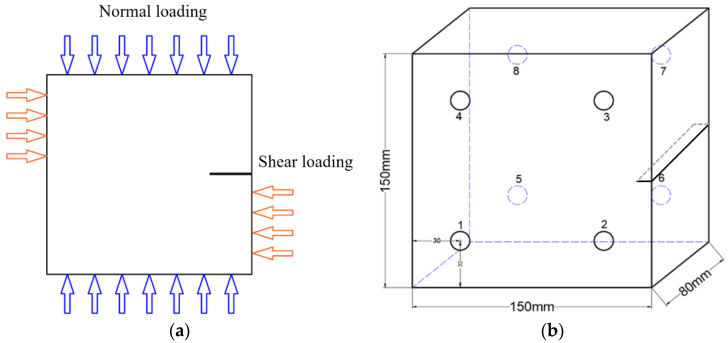
Schematic diagram of the loading mode and the position of AE sensors. (**a**), The loading mode of specimens, the orange arrow in the horizontal direction represents the shear load, and the blue arrow in the vertical direction represents the normal load. (**b**), The position of AE sensors, the circle with number 1–8 represents the position of all AE sensors.

The test load consists of normal loading and shear loading (Figure 1a). To minimize the loss of AE signals and obtain as many AE signals as possible in the center of the rock specimen, AE sensors are arranged in the four corners of the front and back of the specimen (Figure 1b).

**Figure 2:**
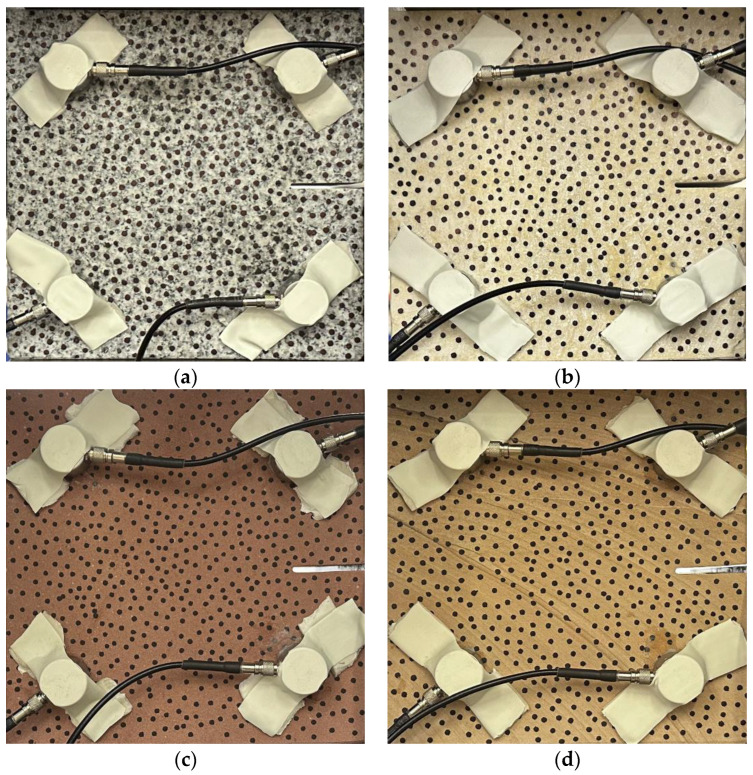
Rock specimen. ((**a**), RSLT-1, granite; (**b**), RSLT-3, white sandstone; (**c**), RSLT-4,red sandstone; (**d**), RSLT-5, yellow Sandstone).

Granite (Figure 2a), white sandstone (Figure 2b), red sandstone (Figure 2c), and yellow sandstone (Figure 2d) are selected as specimens; each rock type has a specimen for shear slip test. The sample numbers corresponding to each rock type are RSLT-1, RSLT-3, RSLT-4, and RSLT-5. Specimen size is 150 × 150 × 80 mm.

A transfixion crack of 20 mm is prefabricated at the 75 mm position in the middle of the right side, parallel to the direction of the shear load. After the experiment, the demonstration debris falling from each rock specimen was sampled for mineral identification experiments. The mineral composition, proportion, and particle size (PS) of four rock specimens are shown by mineral identification tests (Table 1).

The minerals of granite are composed of anorthose, potash feldspar, quartz, amphibole, and mica. White sandstone is composed by quartz, kaolin, gothite, mica, and feldspar. Red sandstone consists of minerals such as quartz, feldspar, kaliglimmer, montmorillonite, and hematite. Yellow sandstone is composed of quartz, mica, kalijarosite, illite, and feldspar.

### 2.2. Test System

**Figure 3:**
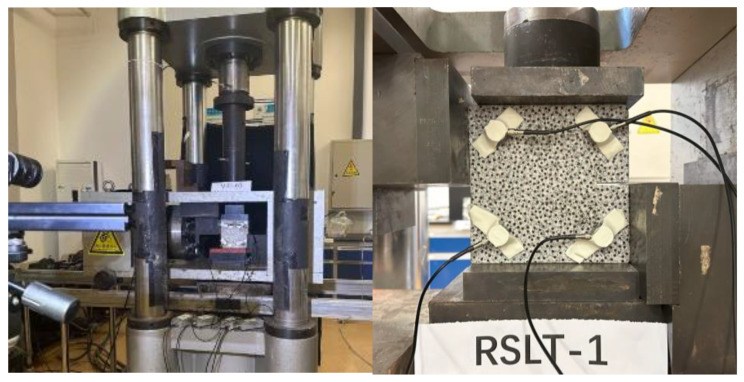
Diagram of test setup.

The test system is shown in Figure 3 and is composed of a loading unit, an AE acquisition system, and a HD camera.

The loading unit utilizes a RLW-3000 shear creep testing machine of Changchun Chaoyang test instrument company in Changchun, China, which provides a maximum axial force of 3000 kN and a maximum shear force of 1000 kN. The AE (acoustic emission) acquisition system is PCI-Express type by PAC of American Physical Acoustics Company (Princeton Junction, NJ, USA), which features 16 channels. The HD camera has an image resolution of up to 2048 pixels × 2048 pixels and its sampling rate ranges from 1 to 1000 framers/s.

It is necessary to ensure that the data from the loading equipment and the monitoring equipment are strictly correlated in time before the rock shear-slip test. All rock specimens adopt the same equipment parameter settings: (1) AE monitoring system, 8 models of Nano-30 sensors symmetrically placed on the front and back sides of the specimen. The sampling rate is 1 MSPS, the threshold value of 45 dB, and the preamplifier gain of 30 dB. In order to collect as many acoustic emission signals as possible, it is necessary to ensure that the rock sample is closely coupled with the sensor. During the test, the elastic tape is used to paste the surface of the rock sample with the sensor, and Vaseline is applied between the sensor and the rock sample. (2) HD camera, sampling frequency is 2 frames/s.

**Figure 4:**
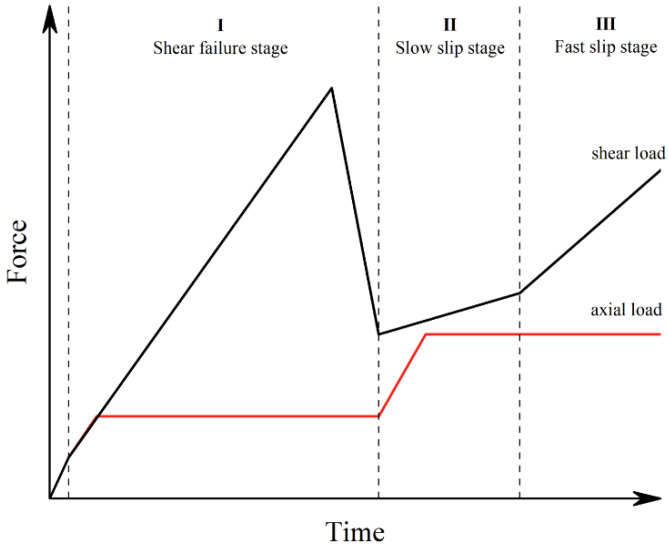
Schematic diagram of normal and shear loading paths.

Tiwari et al. [20] pointed out that the application rate of shear load has a significant effect on the shear mechanics and the fracture properties of rocks. When normal and shear loading reach 50 kN, the shear-slip test begins (Figure 4):

- **I, Shear failure phase**: Loading to 100 kN in the normal direction at a rate of 500 N/s, and at a rate of 0.15 mm/min to shear failure in the shear direction.
- **II, Slow slip phase**: Normal load is to 200 kN at a rate of 500 N/s, and the shear direction of slip magnitude is 1.5 mm at a rate of 0.12 mm/min.
- **III, Fast slip phase**: Normal direction remains unchanged, and the shear direction of slip magnitude is 1.5 mm by 0.3 mm/min. Then, the test is over.

### 2.3. Results

#### 2.3.1. Mechanical Analysis

**Figure 5:**
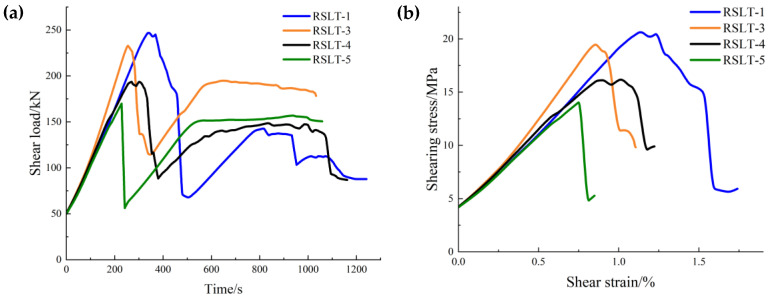
Mechanical curve of shear stress in rock shear-slip test. ((**a**), Shear load-time curve; (**b**), Shear stress-shear strain curve in shear failure phase).

Figure 5a,b depict the load-time curves and the shear stress-strain curves before shear failure, and the shear strain is the deformation of the whole specimen in the shear direction.

Both curves exhibit distinct mechanical characteristics.

- **Granite RSLT-1 specimen:** It exhibits the highest shear strength, the lowest strength in the slip phase, the largest stress drops after the peak, and the largest shear strain in the shear failure phase.
- **White sandstone RSLT-3 specimen:** The shear strength of the specimen closely resembles that of RSLT-1. The strength of the slip phase is significantly higher than that of the other specimens. The stress drop after the peak is the smallest. The shear strain in the shear failure phase is lower than that of the RSLT-1 specimen.
- **Red sandstone RSLT-4 specimen:** The shear and slip phases exhibit characteristics with low values. The difference between the two values is minimal. The shear strain in the shear failure phase is lower than that of the white sandstone RSLT-3 specimen.
- **Yellow sandstone RSLT-5 specimen:** The shear strength is the lowest. The shear failure time is the earliest. The post-peak stress is reduced to the preloading stress level, but the strength of the slip phase is relatively high. The shear strain in the shear failure phase is the smallest.

From the above statistics, the order of shear strength is as follows: RSLT-1 > RSLT-3 > RSLT-4 > RSLT-5. The slip intensities are in the order of RSLT-3 > RSLT-5 > RSLT-4 > RSLT-1. The stress drop of shear strength and the slip strength reflect the energy dissipation when shear failure occurs with the same loading path. The stress drop of granite is the largest, and the energy dissipation of shear failure strain is the largest. The minimum stress drop of white sandstone indicates the smallest energy dissipation of shear failure strain.

#### 2.3.2. Fracture Features

**Figure 6:**
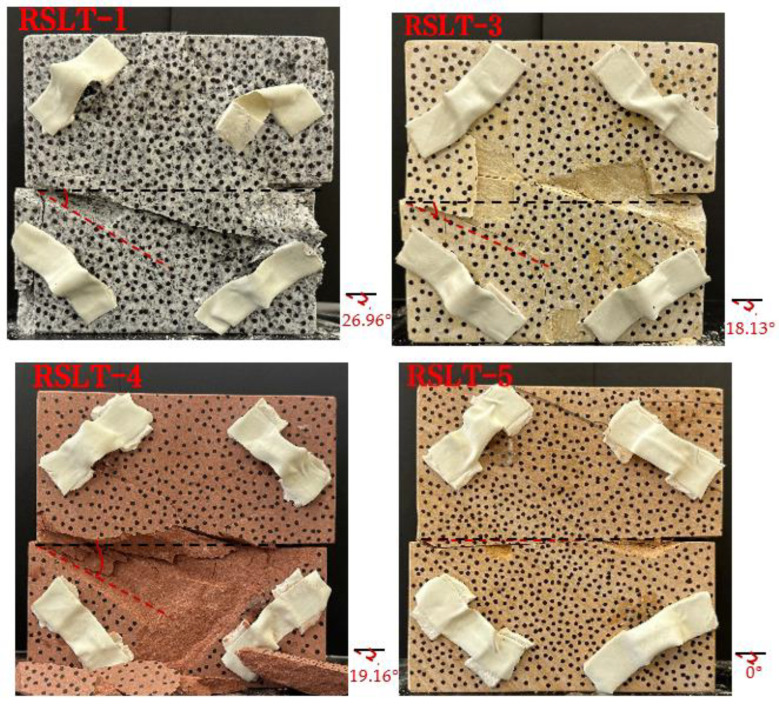
Failure morphology of rock specimens. (RSLT-1 is a granite specimen, RSLT-3 is a white sandstone specimen, RSLT-4 is a red sandstone specimen, RSLT-5 is a yellow sandstone specimen).

Under the same loading conditions, the fracture patterns vary among different types of rock specimens (Figure 6).

A lower shear strength corresponds to a lesser degree of crushing and a reduced deviation of the shear fracture surface from the horizontal plane and the prefabricated fracture. The ultimate failure of the rock specimen is further elaborated below.

- **Granite RSLT-1 specimen:** The dominant crack in this specimen is not parallel to the prefabricated fracture. The dominant crack is at a dip of 26.96° to the shear direction. The secondary crack exhibits outward collapse with tensile fracture, leaving a small crater on the surface of the specimen. Due to the combined effects of axial and shear loads, a significant peeling surface is created, causing the front side to be almost entirely detached from the specimen.
- **White sandstone RSLT-3 specimen:** The shear crack surface of the specimen does not penetrate the prefabricated fracture, and the dominant crack is not parallel to it. The dominant crack is at a dip of 18.13° to the shear direction. The secondary crack is distributed in the middle of the specimen. Some flakes are peeled off, which extend upward from the crack initiated to the upper edge of the specimen. Compression-shear cracks are formed under the combined action of axial and shear loads.
- **Red sandstone RSLT-4 specimen:** The shear crack surface of the specimen penetrates from the prefabricated fracture and the dominant crack surface is not parallel to the prefabricated fracture. The dominant crack is at a dip of 19.16° to the shear direction. Under the development of a secondary crack, a large area of thin spalled slices appears on the surface of rock specimens, with most of the spalled flakes being wedge-shaped.
- **Yellow sandstone RSLT-5 specimen:** The shear crack surface of the specimen extends from the prefabricated fracture, and the primary crack surface is almost parallel to the shear direction. The dominant crack is at a dip of 0° to the shear direction. The rock specimen exhibits minimal fragmentation, developing only a shear fracture surface near the prefabricated fracture. Additionally, there are a few cracks along the intrinsic bedding plane of the specimen at this site, and only a few rock blocks have dislodged.

Table 2 statistics on the crack morphology of rock specimens during various loading phases.

The red line represents a crack, the line thickness denotes the dominant and secondary cracks, and the red shaded area illustrates the fractured area that emerges after the rock flakes spall or debris falls. The evolution of crack development follows the summarized law as follows.

- **Granite RSLT-1 specimen:** The macro-scale crack does not penetrate from the prefabricated crack but expands obliquely downward at a certain dip. The continuous compression at the loading end near the prefabricated crack forms a fracturing area and a small number of rock fragments. Towards the end of the slow slip phase, a new crack is initiated by the peeling of thin sections on the rock surface due to the expansion of the rock specimen. As the rapid slip phase concludes, a dominant crack develops a branch obliquely to the upper left due to mutual friction, and a vertical tensile fracture emerges in the lower left corner of the specimen.
- **White sandstone RSLT-3 specimens:** The crack initiation position is closely related to the prefabricated crack. At the end of the slow slip phase, cracks that originate at the prefabricated crack develop horizontally to the left, and a large number of cracks are distributed in the shear crack zone. At the end of the rapid slip phase, a new shear crack parallel to the dominant crack is formed at the contact position on the left side of the specimen. A small amount of rock flakes peels off at the bottom of the specimen.
- **Red sandstone RSLT-4 specimen:** The rock specimen originates from the dominant crack, and several secondary cracks are formed. The secondary cracks extend and are arranged like a plume. At the end of the slow slip phase, the secondary crack expands, and a small area of rock flake spalling occurs. At the end of the rapid slip phase, the dominant and secondary cracks cause many fragments. The fragments of the shear zone in the middle of the specimen are wedge-shaped. The secondary cracks in the upper part of the specimen further extend.
- **Yellow sandstone RSLT-5 specimens:** The rock specimen is formed with horizontally dominant cracks. An unopened crack along the rock horizon is formed in the upper part of the shear plane. At the end of the slow slip phase, secondary cracks controlled by layered structures appear near the dominant crack, arranged in a plume. Small-area rock lamellar detachment occurs at the prefabricated opening. At the end of the rapid sliding phase, a small fragment falls from the secondary crack on the left. There is no obvious change in the crack morphology on the surface of the specimen.

#### 2.3.3. The Features of Fracturing Evolution

The features of AE ringing count and cumulative energy reflect the activity of internal fracture events in rock specimens during each loading phase, as well as the associated energy dissipation law.

**Figure 7:**
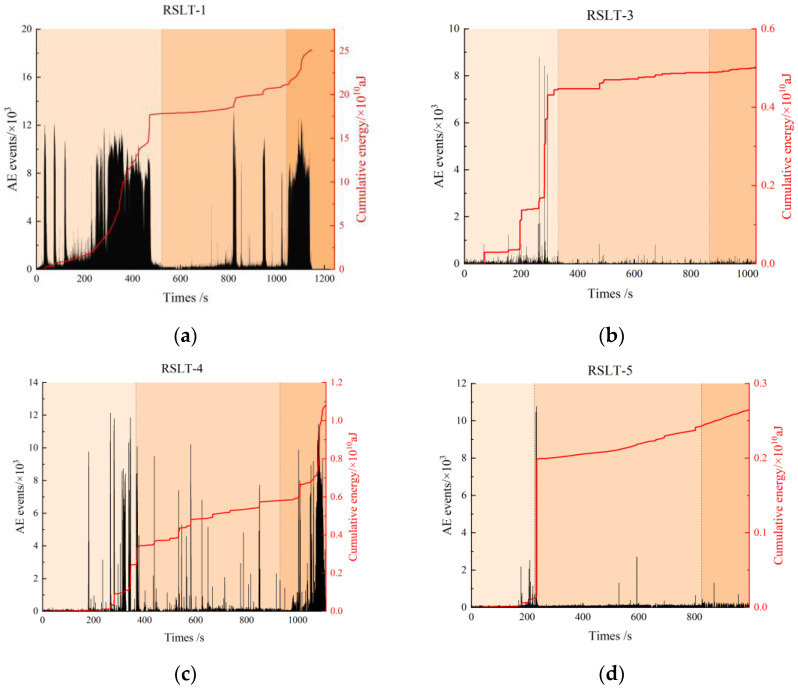
Cumulative AE energy, counting, and time curves of rock fracturing process. ((**a**), RSLT-1; (**b**), RSLT-3; (**c**), RSLT-4; (**d**), RSLT-5).

- **Granite RSLT-1 specimen (Figure 7a)**: In the shear failure phase, the AE activity level is high, and the number of AE events gradually increases. Cumulative AE energy increases slowly in the early and middle phases, and then rapidly in the later phase, reaching 13,000 AE events and 1.75 × 10^12^ aJ of cumulative AE energy. In the slow slip phase, AE events and cumulative AE energy remain at low levels, increasing in a stepwise manner in the later phase. In the rapid slip phase, AE activity increases significantly. As the system approaches final instability, AE activity peaks, with AE events reaching up to 18,000 and cumulative AE energy up to 2.5 × 10^12^ aJ.
- **White sandstone RSLT-3 specimen (Figure 7b):** In the shear failure phase, cumulative AE energy increases in steps during the early and middle phases, while AE events fluctuate at a low level. In the later phase, AE events reach close to 9000, cumulative AE energy increases to 4.5 × 10^9^ aJ. In the slow slip phase, AE events remain at a low level, and accumulated AE energy increases slowly in a step-like manner. In the rapid slip phase, AE events fluctuate at a low level, and the increase in accumulated AE energy slows down further. As the system approaches final instability, cumulative AE energy reaches 5 × 10^9^ aJ.
- **Red sandstone RSLT-4 specimen (Figure 7c):** AE events remain high, and cumulative AE energy increases significantly. AE activity starts at a low level in the early stages of the shear failure phase. Then, AE events increase sharply to 12,000 in the middle and late phases, while cumulative AE energy increases up to 3.5 × 10^9^ aJ in a stepwise manner. During the slow slip phase, AE events decrease slightly, and the step-by-step growth of cumulative AE energy slows down. In the rapid slip phase, AE activity intensifies. As the system approaches final instability, AE events decrease from 12,000 to 4000, and cumulative AE energy reaches up to 1.08 × 10^10^ aJ.
- **Yellow sandstone RSLT-5 specimen (Figure 7d):** In the shear failure phase, AE activity varies greatly. AE events and cumulative AE energy are at a low level in the early and late phases. In the medium term, AE events increase by nearly 11,000, and cumulative AE energy reaches 2.0 × 10^9^ aJ. In the slow slip phase, AE events remain unchanged, while cumulative AE energy increases slowly. In the rapid slip phase, AE activity remains at a similar level as in the previous phase. AE events drop to 500, and cumulative AE energy increases to 2.6 × 10^9^ aJ in the final instability.

Thus, AE activities vary noticeably under the same loading conditions. AE events and accumulated AE energy also fluctuate periodically with the loading phase. It has been noted that the AE energy produced by shear failure exceeds that of tensile failure [10]. During the shear failure phase, the number of micro-shear cracks in the RSLT-1 specimen was significantly higher than in the other specimens.

## 3. The Features of Acoustic Emission (AE) and Energy Dissipation

### 3.1. The Difference of AE Characteristics in Different Loading Phases

RA (Rise Time/Amplitude) and AF (Average Frequency) are often used to reveal the mechanism of rock fracturing. RA and AF values are calculated as,

(1) $$\left\{\begin{array}{l} \mathrm{RA}=\frac{\mathrm{Rise}\;\mathrm{Time}}{\mathrm{Amplitude}}\\ \mathrm{AF}=\frac{\mathrm{Counts}}{\mathrm{Duration}\;\mathrm{Time}} \end{array}\right.$$

The AE waveform of the tensile failure has a shorter rise time and a higher frequency. At this time, the RA value is smaller and the AF value is larger. On the contrary, if the AE waveform signal frequency is low and the rise time is long, it is a shear failure. At this time, the AF value is small and the RA value is large [21]. In general, AE signals characterized by high AF and low RA values correspond to shear type, while AE signals characterized by low AF and high RA values correspond to tensile type [22].

RA/AF value characterizes the fracturing types from qualitative and semi-quantitative aspects. However, quantitative analysis is necessary to determine the energy proportions corresponding to different fracturing types. Combined with the variation law of the corresponding AE parameter characteristics of each loading phase, three fracturing types were distinguished: tensile, shear, and tension-shear composite types. The calculation formulas are as follows:

(2) $$\left\{\begin{array}{l} \theta =\frac{\mathrm{AF}}{\mathrm{RA}}\\ \theta \geq 1000,\;\mathrm{Tenslie}\;\mathrm{crack}\\ 1\leq \theta <1000,\;\text{Tenslie-Shear}\;\mathrm{mixed}\;\mathrm{crack}\\ \theta <1,\;\mathrm{Shear}\;\mathrm{crack} \end{array}\right.$$

**Figure 8:**
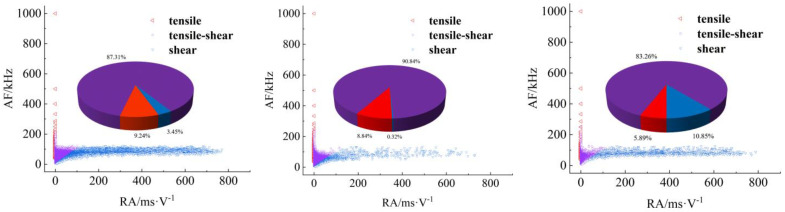
Distribution and proportion of fracturing types and their proportions during the loading process of RSLT-1. (From left to right: shear failure, slow slip, and fast slip phases).

**Figure 9:**
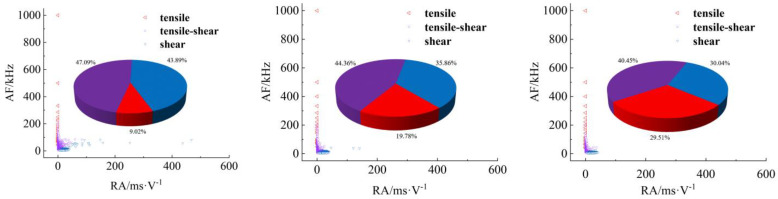
Distribution and proportion of fracturing types and their proportions during the loading process of RSLT-3. (From left to right, shear failure, slow slip, and fast slip phases).

**Figure 10:**
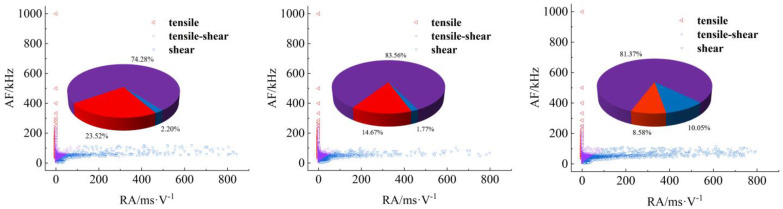
Distribution and proportion of fracturing types and their proportions during the loading process of RSLT-4. (From left to right: shear failure, slow slip, and fast slip phases).

**Figure 11:**
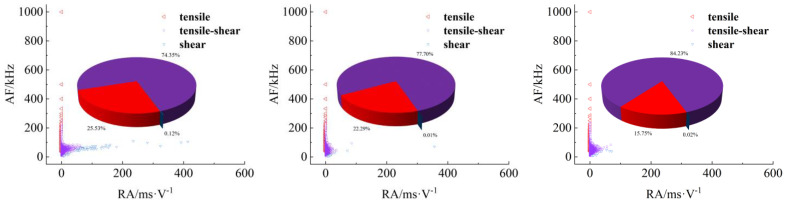
Distribution and proportion of fracturing types and their proportions during the loading process of RSLT-5. (From left to right: shear failure, slow slip, and fast slip phases).

Figure 8, Figure 9, Figure 10 and Figure 11 show AF/RA distribution and the energy proportion of different fracturing types during the shear-slip process.

- **Granite RSLT-1 specimen (Figure 8):** The mainly tensile and shear composite types, accounting for more than 80% of the whole test. Tensile type shows a decreasing trend, and the variation range is “9.24% > 8.84% > 5.89%”. The variation of the three types is “3.45% > 0.32% > 10.85%”, and the shear type in the rapid slip phase increased significantly.
- **White sandstone RSLT-3 specimens (Figure 9)**: The proportion of tension-shear composite type is “47.09% > 44.36% > 40.45%”, showing a slight decreasing trend. Tensile type accounts for “9.02% > 19.78% > 29.51%”, increasing by 10%. Shear type decreases from “43.89% > 35.86% > 30.04%”, which showed a decreasing trend.
- **Red sandstone RSLT-4 specimens (Figure 10):** The mainly tensile and shear composite types, account for “74.28% > 83.56% > 81.37%”. Tensile type accounts for “23.53% > 14.67% > 8.58%”, showing a decreasing trend. The proportion of shear fractures is “2.20% > 1.77% > 10.05%”, and the slow slip phase increased significantly.
- **Yellow sandstone RSLT-5 specimen (Figure 11):** the main failure mode is tension-shear composite, and its proportion increases slightly with the loading process, 74.36% > 77.70% > 84.23%. Tensile type decreases slightly in the first two phases, but decreases significantly in the rapid slip phases, accounting for “25.53% > 22.29% > 15.75%”. The proportion of shear type is very small, accounting for “0.12% > 0.01% > 0.01%”.

The proportion of shear types in granite specimens is significantly higher during the fast slip phase compared to the previous loading phase, consistent with the findings of Liu et al. [23,24]. In white sandstone, red sandstone, and yellow sandstone specimens, the proportion of shear types has decreased over three phases. There are two trends in the proportion of tensile types. Firstly, the tensile type in rock specimens with high shear strength shows a decreasing trend. Secondly, the tensile type in rock specimens with high shear strength increases [25].

### 3.2. Correlation between Fracture Type and Acoustic Emission Energy

Shi et al. [26] pointed out that crack propagation can be summarized by the following sequence: a small number of cracks > isolated cracks clustered near faults > cracks dominated by shear fractures propagate along the faults > tensile cracks connect shear cracks perpendicular to crack propagation.

**Figure 12:**
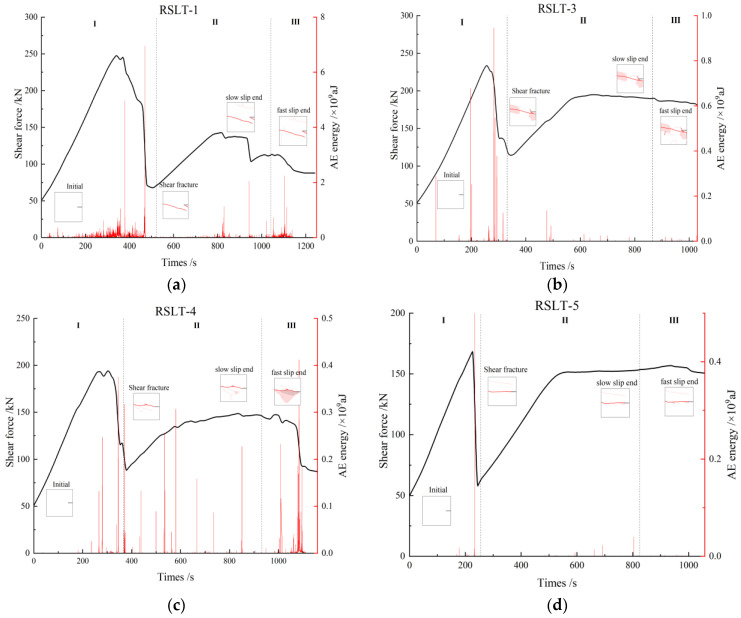
Relationship between shear load, AE energy, fracture propagation, and time during the shear-slip process. (**a**), RSLT-1; (**b**), RSLT-3; (**c**), RSLT-4; (**d**), RSLT-5. I–III in the figure represent the shear failure phase, the slow slip phase and the fast slip phase, respectively.

As shown in Figure 12, crack status has a good correspondence with the curves of AE energy evolution and shear load curve.

The dominant crack formation, the sudden drop of the mechanical curve, the peak AE energy, and the peak AE energy all appear at the step-like decline of the mechanical curve. In the slip phase, the AE energy of granite RSLT-1 specimen and red sandstone RSLT-4 specimen fluctuates obviously, while the AE activity of white sandstone RSLT-3 specimen and yellow sandstone RSLT-5 specimen is weak. The difference in AE energy in the fast slip phase is analyzed from the multi-scale fracturing events.

**Figure 13:**
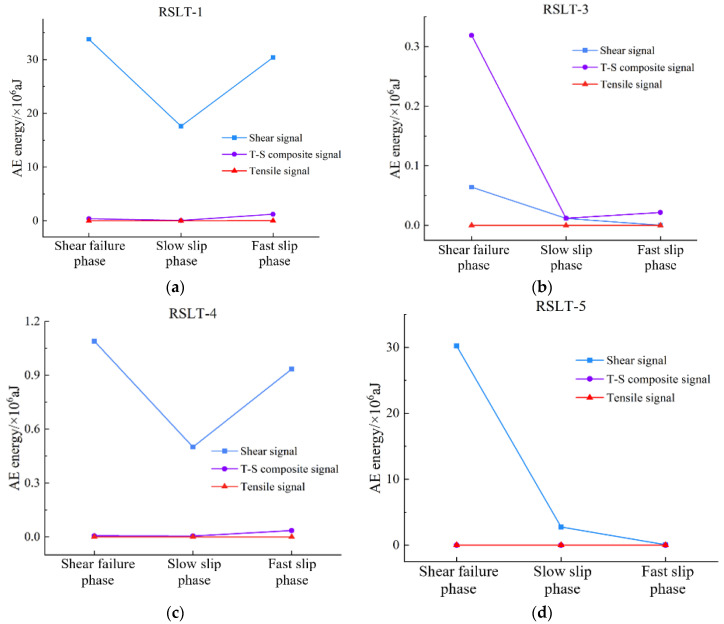
Relationship between fracture type and AE energy level of different rock specimens during the shear-slip process. (**a**), RSLT-1; (**b**), RSLT-3; (**c**), RSLT-4; (**d**), RSLT-5.

The crack is extending from the nearby of the shear plane to both sides. A comparison of AE energy levels for three fracture types is shown in Figure 13.

- **AE energy of shear type:** From shear failure to slow slip phases, RSLT-1 decreases from 3.38 × 10^7^ aJ to 1.76 × 10^7^ aJ, and RSLT-4 is from 1.09 × 10^6^ aJ to 5.0 × 10^5^ aJ. In the fast slip phase, RSLT-1 rises to 3.04 × 10^7^ aJ, and RLST-4 increases to 9.34 × 10^5^ aJ, which is significantly higher than the others. RSLT-3 and RSLT-5 decrease, RSLT-3 decreases from 6.34 × 10^4^ aJ to 1.17 × 10^4^ aJ, then to 3.5 × 10^2^ aJ. RSLT-5 decreases from 3.02 × 10^7^ aJ to 2.76 × 10^6^ aJ, then falls to 7.14 × 10^4^ aJ.
- **AE energy of tensile type:** Tensile signal energy of different rock specimens varies significantly. RLST-1 measures at 10^3^ aJ in shear failure and slow slip phases and increases to 104 aJ in the fast slip phase. RSLT-3 measures less than 10 aJ in all three phases. RSLT-4 is approximately 10^2^ aJ in the shear failure phase and ranges from 10^2^ aJ to 3 × 10^2^ aJ in slow slip and fast slip phases. RSLT-5 shows minimal change, with the entire shear-slip process totaling about 150 aJ.
- **AE energy of tensile-shear (TS) composite type:** The AE energy levels of tensile-shear composite type for RSLT-1 and RSLT-4 are 4.0 × 10^4^ aJ and 6.18 × 10^3^ aJ in the shear failure phase. These values increase significantly to 1.22 × 10^6^ aJ and 3.57 × 10^4^ aJ in the fast slip phase. RSLT-3 and RSLT-5 maintained levels of 10^5^ aJ and 10^3^ aJ, respectively, with no significant changes.

In summary, the energy dissipation of tension-shear composite type ranges from 10^3^ aJ to 10^6^ aJ, shear type ranges from 10^4^ aJ to 10^7^ aJ, and tensile type ranges from 10^1^ aJ to 10^3^ aJ. The ratio of the energy index of the three types is 10:100:1. The difference in energy levels between different fracture types is similar to the results of compression experiments using salt rock [19]. Different rock specimens also exhibit varying AE activity. Granite specimens are notably brittle rocks, displaying strong fragmentation and fracturing features, resulting in complex fracture types and intense AE activities. White sandstone, red sandstone, and yellow sandstone specimens exhibit a relatively dominant crack formation, with a strong correlation between crack propagation and prefabricated cracks, leading to weaker AE activity [27].

## 4. Discussion

**Figure 14:**
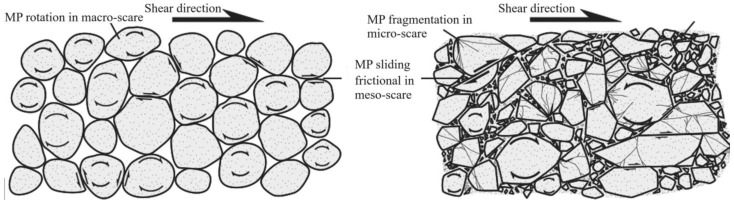
Schematic diagram of macro-meso-micro scale fractures on slip crack surface. The arrows in the figure represent the rotation state of rock particles under horizontal shear load.

In the shear failure phase, the macroscopic fracture of rock samples occurs as a whole, both energy dissipation and stress drop are the largest (Figure 5), and the fracture type is complex (Figure 8, Figure 9, Figure 10 and Figure 11). In fast and slow slip phases, fracture events covering macro-meso-micro scales are concentrated in the dominant crack, such as mineral particle (MP) rotation in the macro-scale, MP sliding frictional in the meso-scale, and MP fragmentation in the micro-scale (Figure 14).

Fracturing in macro- and meso-scales is accompanied by shear AE signals, and in the micro-scale is associated with tensile AE signals. While fractures at three scales occur simultaneously, the AE signal will be characterized by a tension-shear composite type.

Granite is a crystalline rock, while white sandstone, red sandstone, and yellow sandstone are cemented rocks. The descending sequence of mineral particle sizes is granite, white sandstone, red sandstone, and yellow sandstone. The four rock specimens in this study differ in terms of the type, proportion, and size of minerals.

- **Granite specimens:** The minerals include anorthosite, potash feldspar, quartz, amphibole, and mica. The particle size of anorthosite and potassium feldspar varied the most (Table 1). Shear and tensile AE signals are not effectively distinguished, and the tension-shear (TS) composite AE signal accounted for the largest proportion (Figure 11). Fracturing events at macro-meso-micro scales occurred simultaneously (Figure 14).
- **White sandstone specimens:** The minerals include quartz, kaolin, gothite, mica, and feldspar (Table 1). Quartz has the largest particle size and the highest proportion. Goethite has the smallest particle size and the highest hardness. TS composite, shear, and tensile types AE signals are relatively developed in the shear failure phase. The sliding friction between minerals increases as the slip rate increases (Figure 14). The proportion of shear-type AE signals gradually increases (Figure 9).
- **Red sandstone specimens:** The mineral composition includes quartz, feldspar, kaliglimmer, montmorillonite, and hematite (Table 1). The size of each mineral particle ranges from 0.005 mm to 0.5 mm. The shear AE signal increases due to the micro-scale sliding friction of strong and tough minerals (Figure 14). The fragmentation of micro-scale mineral particles leads to an increase in the proportion of tensile-type AE signals (Figure 10).
- **Yellow sandstone specimens:** The minerals include quartz, mica, kalijarosite, illite, and feldspar (Table 1). The size of mineral particles is distributed between 0.0002 mm and 0.2 mm. The rotation of macro-scale mineral particles and the sliding friction between meso-scale mineral particles account for the highest proportion (Figure 14). The proportion of tension-shear composite AE signals is the highest (Figure 11).

The size of mineral particles significantly influences the failure mode of microcracks within rocks and the evolution of macroscopic fractures on their surfaces [28]. Changes in mechanical properties and acoustic emission characteristics reflect differences in rock microstructure at the macroscale level [29]. In short, the energy dissipation of rock fracturing is closely related to the fracture type under the same loading conditions. Mineral composition, particle size, and diagenetic mode are the main mechanisms of energy dissipation in each rock [30]. Shear signal predominates in shear failure and fast slip phases (Figure 13). The TS composite type is the primary event, with relatively low energy dissipation in the slow slip and fast slip phases (Figure 13).

## 5. Conclusions

This experimental study explores the failure characteristics of rocks during shear slip processes from the perspectives of acoustic emission characteristics and energy, provides new evidence and physical interpretation for revealing the types of fractures at different stages of shear slip and the energy dissipation levels of different types of fractures, and the following conclusions can be drawn from this study:

1. There is a strong correlation between fracture type, energy dissipation, and AE features. The more complex the fracture type, the stronger the energy dissipation, and the more intense the AE activity. The largest proportion of energy dissipation in granite specimens is the TS composite type. In other rock specimens, the fracture type is shear in shear failure, TS composite in the slow slip phase, and tensile in the fast slip phase.
2. In the whole process of rock shear-slip, the ratio of energy index of TS composite, shear, and tensile types is 10:100:1. Different rock specimens have varying features of hardness and brittleness, which also results in different AE activity during fracturing.
3. The energy dissipation of rock fracturing is discussed in terms of mineral composition, particle size, and diagenetic mode. The high energy dissipation of granite specimens is primarily dominated by the shear type, while the significant energy dissipation of the other three specimens is dominated by the TS composite type.

There are still some difficulties and challenges in the research of rock shear slip failure processes. It is encouraged to further investigate from the perspectives of cross-scale evolution of fractures and microstructure of minerals inside rocks.

## Figures and Tables

**Table 1:** Statistical table of mineral type, proportion, and particle size (PS) of different rock specimens.

Number of Rock Specimens	Mineral Type	Proportion/%	Particle Size/mm
RSLT-1 (granite)	Anorthose	38	0.5~3
	Potash feldspar	30	0.5~5
	Quartz	25	1.5
	Amphibole	4	0.6
	Mica	3	1
RSLT-3 (white sandstone)	Quartz	50	2~4
	Kaolin	20	0.12
	Gothite	18	0.09
	Mica	8	0.5
	Feldspar	4	0.3
RSLT-4, (red sandstone)	Quartz	50	0.1~0.5
	Feldspar	25	0.05~0.5
	Kaliglimmer	10	0.05~0.5
	Montmorillonite	10	0.1
	Hematite	5	0.06
RSLT-5 (yellow sandstone)	Quartz	55	0.1~0.15
	Mica	25	0.02~0.08
	Kalijarosite	10	0.002
	Illite	5	0.0002
	Feldspar	5	0.2

**Table 2:** Crack develops morphology of rock specimens during different loading phases.

No.	Initial Phase	Shear Failure Phase	Slow Slip Phase	Fast Slip Phase
RSLT-1	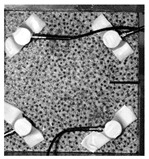	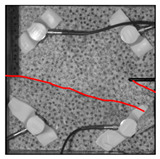	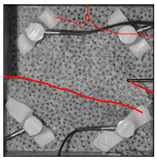	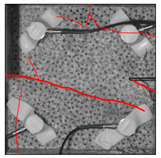
RSLT-3	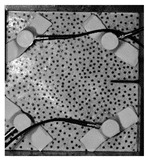	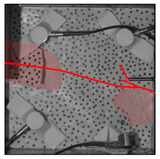	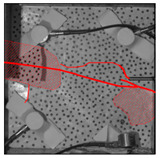	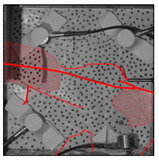
RSLT-4	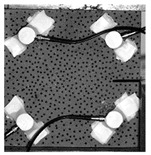	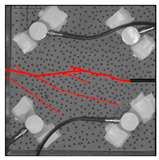	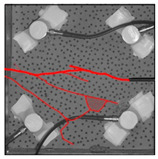	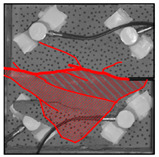
RSLT-5	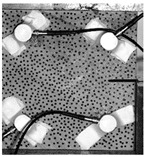	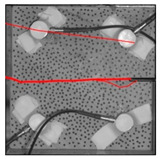	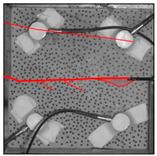	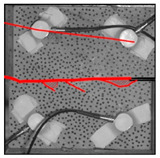

## Data Availability

The original contributions presented in the study are included in the article, further inquiries can be directed to the corresponding author.
